# Role for TREK-1 as a polymodal sensor and regulator of cell activity

**DOI:** 10.1080/19336950.2026.2675071

**Published:** 2026-05-19

**Authors:** Alexander J. Winkle, Aparna Odayil Muralidharan, Christian Renee McClenney, Kamellia Karimpour, Taukeed Muhammad Ashfaque, Shaihrum Qureshi, Thomas J. Hund, Drew Nassal

**Affiliations:** a Frick Center for Heart Failure and Arrhythmia, Dorothy M. Davis Heart and Lung Research Institute, The Ohio State University, Columbus, OH, USA; b Department of Biomedical Engineering, College of Engineering, The Ohio State University, Columbus, OH, USA; c Division of Cardiovascular Medicine, Department of Internal Medicine, College of Medicine, The Ohio State University, Columbus, OH, USA; d Department of Physiology & Cell Biology, College of Medicine, The Ohio State University, Columbus, OH, USA

**Keywords:** TREK-1, arrhythmia, depression, heart failure, therapeutic target

## Abstract

TREK-1 (KCNK2) is a polymodal two-pore domain potassium (K2P) channel that functions as a background K^+^ conductance and integrator of mechanical, chemical, and thermal stimuli across diverse cell types. Its unique structure enables sensitivity to membrane stretch, lipid composition, pH, temperature, pharmacologic agents, and intracellular signaling pathways. Beyond shaping resting membrane potential and excitability, TREK-1 engages in noncanonical signaling roles involving protein–protein interactions, trafficking, and modulation of intracellular signaling cascades such as MAPK and calcineurin pathways. TREK-1 is widely expressed in the nervous system, where it regulates neuronal firing, pain sensitivity, mood, and neuroprotection. In the heart, TREK-1 influences action potential duration, mechano-electric feedback, sinoatrial node function, and stress-induced remodeling, with mutations linked to arrhythmogenesis. In fibroblasts and fibroblast-like cells, TREK-1 acts as a mechanotransducer driving differentiation and fibrosis through MAPK signaling. TREK-1 also modulates immune activation, inflammasome signaling, adipogenesis, epithelial injury responses, vascular tone, and cancer cell proliferation. Across tissues, dysregulation of TREK-1 contributes to pathological excitability, fibrosis, inflammation, and degeneration. Given its multimodal regulation and broad impact on cellular function, TREK-1 represents a compelling therapeutic target, though challenges remain due to limited subtype-selective pharmacology and incomplete understanding of its nonionic signaling roles.

## Intro

### Discovery/Classification of K2P channels

**Figure 1.**
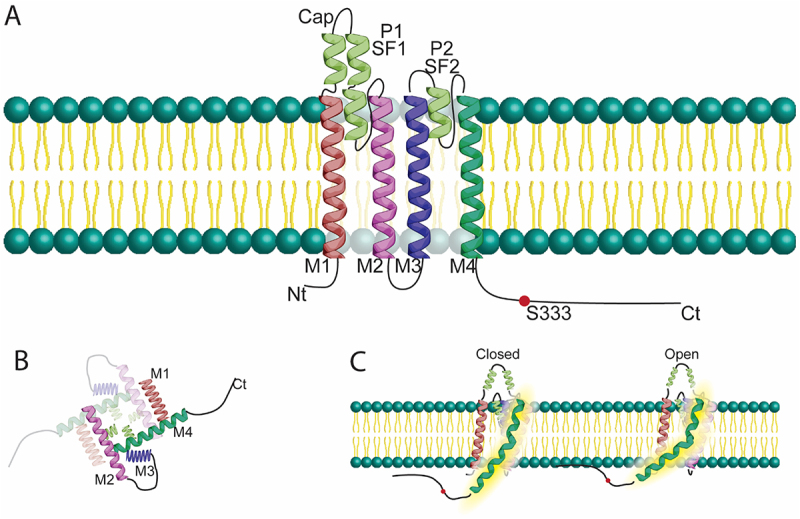
A. A schematic drawing of TREK–1 in the membrane showing cap, selectivity filter, and transmembrane domains, color-coded for identification. B. Dimerized TREK–1 illustrated from the intracellular vantage point, showing the central pore structure. As α–helices, M1 and M3, share the same pitch and diameter as M2 and M4, but are shown compressed to highlight their angle relative to the viewing plane. C. Illustration of structural diffrences between open and closed states, highlighting the positional change of the M4 domain with glycine hinge function and proximity of the C terminal tail to the membrane subsurface. (P1/2: pore 1/2 domains, SF1/2: selectivity filter 1/2, M1–4: transmembrane helix 1–4, Nt: N–terminus, Ct: C–terminus).

Potassium (K^+^) channels are ubiquitous membrane proteins essential for regulating cellular excitability and electrolyte homeostasis [1]. They are encoded for by at least 75 genes in mammals, and exhibit remarkable structural and functional diversity that distinguishes them from other ion channel families [2]. Unlike voltage-gated sodium (Na_v_) and calcium (Ca_v_) channels, which are each represented by fewer than 10 genes, K^+^ channels comprise multiple superfamilies with distinct architectures and regulatory mechanisms, making them the most diverse group of membrane-associated ion channels [3]. While voltage-gated (K_v_) and inwardly rectifying (K_ir_) potassium channels have long been characterized, a distinct superfamily of K^+^ channels known as two-pore domain potassium (K_2P_) channels was more recently identified [4]. Unlike traditional K^+^ channels, which typically assemble as tetramers of subunits containing one pore domain, K_2P_ channels function as homo- or heterodimers with each subunit consisting of two pore-forming domains (P1 and P2) arranged in tandem, and four transmembrane segments (M1–M4) [5,6]. (Figure 1(A)) Quaternary structure then yields a pseudo-tetrameric form equivalent to traditional K^+^ channels (Figure 1(B))

The TWIK-related K^+^ channel 1 (TREK-1), encoded by the *KCNK2* gene, was one of the first mammalian members of the 15 K_2P_ channel subfamilies to be cloned and characterized [7,8]. TREK-1 belongs to the TREK subfamily, which also includes the TWIK-related K^+^ channel 2 (TREK-2, *KCNK10*) and the TWIK-related arachidonic acid-activated K^+^ channel (TRAAK, *KCNK4*). These channels are distinct from traditional K^+^ channels in that they provide “background” or “leak” K^+^ currents that are voltage-dependent, but not strictly voltage inactivated like K_v_ channels. While K_2P_ channels demonstrate weak voltage-dependence, this is through a complex mechanism that is desensitized by other stimuli such as arachidonic acid (AA) and phosphatidylinositol bisphosphate (PIP2) [9–11] allowing them to pass current across the physiological voltage range [5]. As a result of this, they play a critical role in stabilizing the resting membrane potential (RMP) and influencing action potential duration (APD) and cell excitability [6,12]. As we will further discuss, the functional role of TREK-1 extends beyond its ion flux, as it engages with structural and signaling pathways that dictate cellular and tissue level changes.

The following discussion constitutes a brief introduction to the structure and electrophysiological properties of TREK-1 for the purpose of giving context to later discussion of the channel’s involvement in signaling and disease processes. Much more is known about the structure and function of the channel than is within the scope of this review. For more comprehensive discussions of the channel’s structure and electrophysiological behavior, the reader is directed to the following articles [1,5,8,9,13].

### Structure

As is predicted by the notion that structure begets function, the unique arrangement of TREK-1 underpins its functional properties. The channel assembles as a dimer, with the two pore domains of each subunit combining to form the K^+^ selectivity filter [1,10,14]. The conserved structure of the K_2P_ family monomers leads to heterodimerization, increasing the diversity of channel behaviors [8]. A distinctive feature of K_2P_ channel architecture is the presence of a large extracellular loop between the M1 and P1 domains, referred to as the “cap” domain [15,16]. This extracellular cap allows K^+^ ions to flow through side fenestrations and creates a protected environment around the channel pore perhaps contributing to the difficulty in identifying a selective blocking agent for this channel [17,18]. The structure of the K_2P_ pore is also responsible for the channel’s high selectivity for K^+^ under normal conditions, but notably this selectivity is altered with hypokalemia or extracellular acidosis [17,19]. The channel possesses an intracellular C-terminal domain in differential proximity to the intracellular surface of the membrane between open and closed channel states. This tail acts as an important regulatory hub, influencing the channel’s response to various stimuli [1,13]. Deletion or modification of the C-terminal domain profoundly alters the channel’s sensitivity to physical and chemical modulators, such as stretch, temperature, pH and lipids [5,20]. Structural study in the related K_2P_ member TREK-2 has identified this conformational change is related to a K_2P_ conserved “glycine hinge” leading to a kink in the M4 domain [16,21] (Figure 1(C)) More recent study of the differential gating between TREK-1 and TREK-2 has suggested that this M4 kink mechanism may be insensitive to extracellular stimuli in TREK-1 specifically, and further study is necessary [22]. Furthermore, TREK-1 expression and trafficking are regulated by specific protein-protein interactions. For instance, the channel associates with the cytoskeletal protein β_IV_-spectrin at the cardiac intercalated disc, an interaction required for proper membrane targeting [23]. Additionally, TREK-1 interacts with A-kinase anchoring protein 150 (AKAP150) and microtubule-associated proteins, which modulate its regulation and surface density [5,24].

### Electrophysiological properties

**Figure 2.**
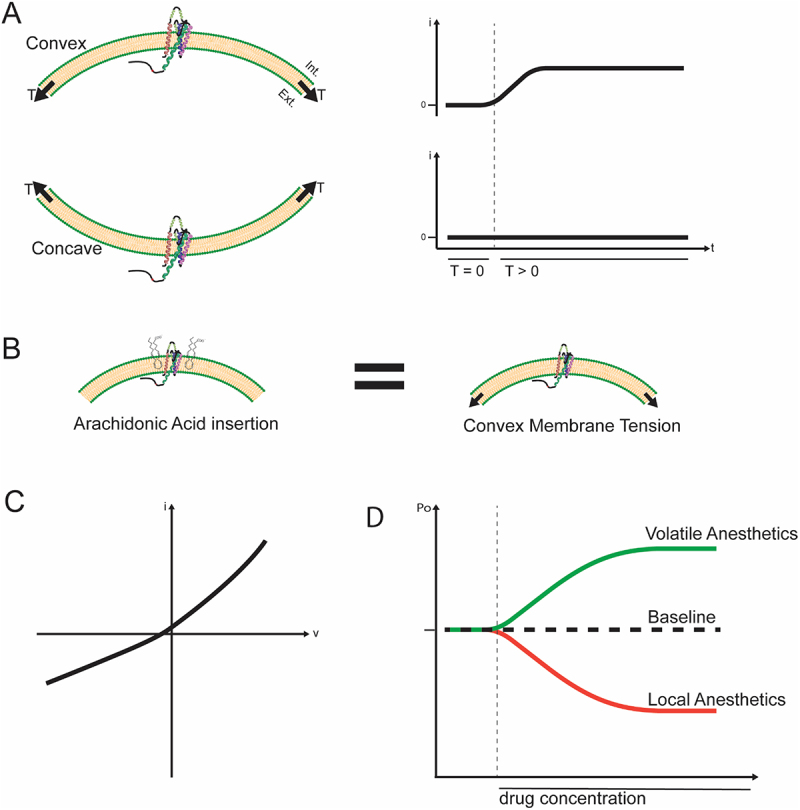
A. Illustration showing selective mechanosensitivity of TREK-1 related K_2P_ member TRAAK in the membrane. The channel is preferentially activated by membrane tension in the convex orientation. B. Illustration showing the mechanism of arachidonic acid (AA) activation of TREK-1. Insertion of (AA) into the membrane is believed to create convex membrane curvature that mimics the effect of membrane tension. C. Cartoonized IV curve showing weak rectifier behavior of TREK-1 in conditions of symmetrical high [K^+^]. D. Effect of different anesthetic classes on the open probability (Po) of TREK-1.

As a mechano-gated channel, TREK-1 is sensitive to membrane stretch, with membrane tension increasing channel open probability. This response is reversible, and study in the related K_2P_ family member TRAAK has demonstrated an activation profile that is more strongly associated with convex membrane curvature than concave or linear stretch [25,26]. (Figure 2(A)) The channel interacts with both the cell membrane and the actin cytoskeleton with deletion/interruption of the cytoskeleton leading to increased channel expression/activity [27]. Progressive deletion of the C-terminus decreases stretch sensitivity, showing the importance of this domain for the channel’s mechano-regulation [5,28]. This C-terminal domain contains two serine residues (S300, S333) that modify channel activity when phosphorylated by Protein Kinase A (PKA) or Protein Kinase C (PKC) [5,24]. Combinatorial experiments using phosphomimetic and phosphoablated mutations demonstrate cooperative behavior between these sites such that maximal channel inhibition is achieved with phosphorylation of both residues [29]. This differential activity is insensitive to modality of activation, and is enhanced by binding of A-kinase anchoring protein 150 (AKAP150) to TREK-1 [5,24].

TREK-1 is also activated by polyunsaturated fatty acids (PUFAs), including arachidonic acid (AA) and lysophospholipids [30,31]. This activation is believed to result from the same mechanism as the mechanosensitivity where insertion of the fatty acids causes conformational changes in the membrane and induces channel opening [1]. (Figure 2(B)) TREK-1 is also activated by intracellular acidification [32]. Modulation of channel activity by mechanical/PUFA activation and pH sensitivity are related to the proximity of the C-terminal domain to the membrane subsurface, with interaction between these two components increasing channel open probability [1,5]. Further, TREK-1 current is susceptible to thermoregulation, with channel inactivation occurring at temperatures below the physiological range [33]. Recent work investigating the thermosensitive properties of the TREK/TRAAK K_2P_ subfamily has identified a temperature response element (TRE) which requires cytoskeletal attachment and is disabled by phosphorylation by PKA at S333 [34].

Due to its unique structure, TREK-1 demonstrates very interesting and complex electrophysiological properties despite the absence of strong voltage dependence. In physiological K^+^ gradients, TREK-1 behaves as an outwardly rectifying background channel [5]. The currents are generally described as instantaneous and non-inactivating, although they show weak voltage dependence [32]. (Figure 2(C)) Unlike classical K_v_ channels, TREK-1 lacks a discrete voltage-sensing domain [35].

Perhaps the most defining feature of TREK-1 is its regulation by a diverse array of both physical and chemical stimuli, enabling it to function as a molecular sensor [30]. Notably, TREK-1 is insensitive to traditional potassium channel blockers including tetraethylammonium (TEA), 4-aminopyridine (4-AP), and low concentrations of barium (Ba^2+^) and cesium (Cs^+^), agents that typically inhibit K_v_ and K_ir_ channels [36,37]. However, TREK-1 is inhibited by local anesthetics lidocaine and tetracaine, as well as the antidepressant fluoxetine [1,37,38]. Conversely, volatile anesthetics such as isoflurane, halothane, and chloroform are reported to activate the channel [25]. (Figure 2(D))

At the single-channel level, TREK-1 exhibits “flickery” burst kinetics consisting of extremely short interruptions during open periods, and long closures between open “bursts” [39,40]. Interestingly, TREK-1 appears to operate in different conductance modes. In heterologous expression systems and native cardiomyocytes, a “large” conductance mode (approximately 100–130 pS in symmetrical high K^+^) and a “small” conductance mode (approximately 40 pS) have been observed [32,39,40]. These variations suggest that the channel may undergo conformational changes or post-translational modifications that alter its pore properties in situ [39].

While highly selective for K^+^ under baseline conditions, TREK-1, and other K_2P_ channels demonstrate altered ion selectivity under specific conditions. As previously mentioned, the selectivity filter (SF) undergoes changes in physiological extremes allowing Na^+^ flux [36,41]. Additionally, mutations and splice variants that affect the SF are reported in TREK-1 and other K_2P_ members and have been linked to electrophysiological pathology in multiple cell types and disease contexts [17,42].

The broad scope of regulatory actors for TREK-1 and other K_2P_ channels underscores the importance of maintaining balanced activation of these channels, as well as their potential for pathological involvement when this regulation goes awry. This includes both expression in traditional electrophysiologic cells such as neurons and myocytes, as well regulatory roles in other cell types not traditionally viewed as being electrophysiologically regulated, including fibroblasts, immune cells, and adipocytes. The diversity of K_2P_ channel subtypes, modes of activation, and patterns of expression results in a broad range of physiological implications across tissue types and organ systems.

## Role of TREK-1 in neurons

TREK-1 is highly expressed in many parts of the central nervous system (CNS) and is vastly interconnected with the homeostatic activity of neuronal excitability and maintenance of the membrane resting potential [43,44]. Alterations in TREK-1 activity have been correlated with multiple conditions such as epilepsy, and ischemic diseases, accentuating the role of TREK-1 in neurological diseases [45,46]. As a background K^+^ channel, TREK-1 sets the resting membrane potential and opposes depolarization [46]. The multimodal sensitivity of TREK-1 to physical stimuli such as membrane stretch, temperature, pH, arachidonic acid, and stretch provides novel opportunities for research in neurons [11,47–49]. Dysregulation of TREK-1 has been linked to altered physiology throughout the brain. In the CNS, the expression of TREK-1 is important for the astrocytic regulation of glutamate, a neurotransmitter responsible for regulating neuronal excitability [50,51]. Consequently, hypersensitivity to a variety of pain stimuli is observed in mouse models lacking TREK-1, where small sensory neurons function abnormally in the knockout genotype [45]. Deletion of TREK-1 demonstrates an anti-depressive phenotype, suggesting a potential relationship and possible pharmaceutical use as an antidepressant [52]. Potentially related to these findings, TREK−1 contributes to the background “leak” current responsible for establishing the neuronal resting membrane potential [50]. By stabilizing the resting potential, TREK-1 acts as a “brake” on neuronal firing. Loss or inhibition of TREK-1 leads to membrane depolarization and enhanced excitability, exhibiting increased action-potential firing in hippocampal CA1 neurons with genetic or pharmacological TREK-1 inhibition [6,50]. However, chronic loss of TREK-1 also disrupts long-term potentiation (LTP) and impairs hippocampal-dependent memory, suggesting that prolonged suppression of TREK-1 must be balanced to preserve cognitive function [1]. In essence, loss of TREK-1 alters neurons to be more excitable, with prolonged excitation potentially leading to decreased cognitive function.

In addition to general cognition, TREK-1 has been implicated in more fundamental neurological processes such as circadian regulation. Giannoni-Guzmán et al. revealed that environmental light exposure alters TREK-1 function and expression in DRN serotonergic neurons via the melatonin receptor 1 (MT-1) [53]. Mice raised under long-day (summer-like) photoperiods had reduced TREK-1 expression (Kcnk2 mRNA) and diminished channel function, resulting in heightened serotonin signaling, a state consistent with antidepressant-like physiology. These photoperiod effects were abrogated in MT-1 knockout mice, indicating that melatonin signaling is required for this plasticity. In contrast, the related K_2P_ channel TASK-1 regulated excitability but was not photoperiod-sensitive, underscoring the specific role of TREK-1 in linking circadian light cues, melatonin signaling, and mood regulation [53,54]. The thermosensitivity of TREK-1 allows control of neuron excitability, making it a compelling target for mood disorders.

TREK-1 is modulated by a variety of common neuropharmacological agents. While some of the most potent are discussed here, a more comprehensive summary is presented in the focused review by Djillani et al. [55] Esketamine, an NMDA-receptor antagonist antidepressant, has been found to alleviate postoperative depression in breast cancer patients and in rodent models [56]. In addition to its role as an NMDA agonist, esketamine has been found to inhibit expression of TREK-1 in hippocampal neurons [56]. Esketamine treatment decreased TREK-1 expression, increased neuronal viability, reduced apoptosis, and restored GABAergic tone, leading to improved depressive behavior, while overexpression of TREK-1 in esketamine-treated rats reversed these anti-depressive behaviors [56]. In patients, esketamine significantly lowered the Hamilton Depression Scale scores at six months, suggesting that, in-part, TREK-1 modulation contributes to durable antidepressant efficacy. These findings point to TREK-1 inhibition as a potential mechanistic component of antidepressant effects in stress- and surgery-related mood disorders [19,56]. On the cellular level, esketamine-treated hippocampal neurons isolated from rats subjected to a depression model revealed differentially expressed genes compared to control [57]. These altered neural genes were found to enrich biological responses such as fear, behavioral defense, cellular components such as neuron projection terminus, axon terminus, and ion channel complex, and molecular functions such as neurotransmitter receptor activity, acetylcholine receptor binding, and gated channel activity [57]. While TREK-1 overexpression restored depression symptoms, the authors did not include a TREK-1 overexpression condition in their differential expression analysis. Therefore it cannot be said whether the differential expression is related to esketamine’s action on TREK-1. Regardless, the demonstrated effect of TREK-1 modulation by esketamine provides novel strategies to alter neural activity and function. Fluoxetine, a commonly prescribed selective serotonin reuptake inhibitor (SSRI) is metabolized in the liver to norfluoxetine, both of which have been found to inhibit TREK-1, although this effect is not part of the canonical SSRI mechanism [40,52,58]. Spadin, an endogenous peptide derived from the sortilin propeptide was discovered to inhibit TREK-1 and increase serotonergic neuron firing, similar to the effects seen in the previously-discussed light exposure study [52,53,59]. Further targeted study has been aimed at modifying spadin to improve pharmacokinetic properties for antidepressant effect, but these modifications have not yet led to success in clinical trials [60–62].

While inhibition of TREK-1 is beneficial for mood regulation, activation of TREK-1 appears therapeutic in degenerative contexts, providing further evidence for the importance of balanced regulation. In the senescence-accelerated SAMP8 mouse model of Alzheimer’s disease, TREK-1 expression declines with age, correlating with elevated glutamate, tau pathology, and cognitive impairment [50]. Treatment with α-linolenic acid, a TREK-1 activator improved hippocampal neuron and astrocyte survival, reduced glutamate accumulation, increased GLT-1 transporter expression, and enhanced learning and memory [63]. These results reveal that TREK-1 activation supports glutamate homeostasis and cognitive function, showing its dual role in excitability control and neuroprotection. Similarly, TREK-1 activation also exerts protective effects in neuropathic pain conditions linked to metabolic stress. In prediabetic models, overexpression of the sorting receptor sortilin in dorsal root ganglion (DRG) neurons promotes internalization and loss of TREK-1 and TREK-2 potassium channels from the plasma membrane [50]. This reduction in background K^+^ conductance enhances neuronal excitability, leading to pain hypersensitivity. Prediabetic rats exhibit mechanical allodynia, thermal hyperalgesia, and downregulated TREK-1/2 expression, whereas genetic suppression of sortilin or pharmacological activation of TREK channels with BL1249 normalizes channel expression, restores neuronal firing thresholds, and alleviates pain behaviors [64]. Similar to its neuroprotective role in Alzheimer’s models, TREK-1 activation counteracts neuronal hyperexcitability and dysfunction in peripheral sensory neurons, highlighting its broader therapeutic potential across both central and peripheral neuropathologies.

## TREK-1 function in cardiac myocytes

TREK-1 plays diverse roles in the cardiac myocyte. Operating as a sarcolemmal ion channel, it is responsible for a small repolarizing leak current with the capacity to regulate cellular excitability, resting membrane potential, and action potential duration (APD) [32]. These channels are found in both atrial and ventricular myocytes, as well as in sinoatrial node (SAN) cells.

Perhaps due to its structural homology with other K_2P_ channels, TREK-1 has proven to be a difficult ion channel to study in isolation. It is modulated by numerous factors, and complete and selective block of the pore has proven elusive [19]. The literature includes many pharmacologic inhibitors of the channel, however these almost universally affect other channels. This may be due to their conserved structure, or as an effect of TREK-1’s propensity to interact with other proteins at the membrane [9]. These nonspecific blockers are still used in study due to lack of pharmacologic alternatives. Other strategies include genetic modulation by gene silencing or knockout models. Development of these models is more costly and time consuming, so these studies are less represented in the literature.

Study in rat ventricular myocytes using the computationally-assisted dynamic clamp predicts that the deletion or subtraction of TREK-1 currents results in a substantial prolongation of APD (e.g. APD_50_ and APD_90_ increased by about 12% in rat ventricular muscle) [65]. By supplementing K^+^ flux during the repolarization phase, TREK-1 channels reduce the time needed for cell repolarization, facilitating Ca^2+^ clearance by shortening APD [66]. The loss of TREK-1 in isolated myocytes was found to prolong APD, delaying transient decay, and suggesting TREK-1 may be important in regulation of Ca^2+^ clearance in conditions of cellular stress. Other recent work has linked TREK-1 activity to Ca^2+^ regulation via its interaction with AKAP5 and calcineurin [67]. TREK-1 was found to be activated by intracellular Ca^2+^ in a manner dependent on the presence of calcineurin. This interaction was strengthened by coexpression with AKAP5 [67], the structural link between TREK-1 and calcineurin [24,68].

As previously discussed, TREK-1 is susceptible to changes in its SF, with potential for pathological outcomes. A patient with right ventricular outflow tract tachycardia (RVOT-VT) generally an idiopathic condition was found to have a single point mutation in the SF of TREK-1 [17,69]. Expression of this mutant TREK-1 (I267T) in xenopus oocytes leads to a reduction of outward current and depolarization of the membrane potential when compared to WT TREK-1. This finding links TREK-1 with other K_2P_ channels known to exhibit pathological mutations and again highlights the importance of these regulators [70–72]. TREK-1 is highly expressed in the SAN, consistent with a critical role in cardiac rhythm [8,73]. Cardiac-specific deletion of TREK-1 in mice leads to altered SAN myocyte excitability and increased incidence of sinus pause in response to adrenergic stress compared to WT [74]. In the embryonic heart, TREK-like currents have an essential role in determining the resting membrane potential (RMP) in atrial myocytes where the primary inward rectifier current I_k1_ is expressed at lower levels compared to ventricular myocytes [38].

TREK-1 is stretch-activated and is expressed in longitudinal bands along the sarcolemma [8,39]. This orientation optimizes the sensor for longitudinal stress response much like a strain gauge. Kamkin et al describe the effect of a non-inactivating stretch activated current (I_SAC_) which increases with hypertrophy, indicating these stretch-activated cationic currents may be important for potassium homeostasis [75]. Importantly, while changes in TREK-1 are reflected in I_SAC_, this current does not differentiate TREK-1 from other stretch-activated cation channels such as TREK-2, TRAAK, and TASK-2 which might also contribute to these observed effects. Without considering I_SAC_ specifically, Wang et al. demonstrated changes in TREK-1 expression, and a TREK-1-like current subsequent to an isoproterenol-induced hypertrophy model, consistent with these findings [32].

TREK-1 activity is tightly coordinated by interaction with other proteins in the submembrane space. β_IV_ spectrin is required for appropriate membrane targeting and activity of TREK-1 in the heart [23]. Mice expressing truncated β_IV_-spectrin lacking interaction with TREK-1 exhibit aberrant localization of TREK-1 in cardiac myocytes as well as decreased activity, leading to delayed action potential repolarization and arrhythmia. Further, Popeye domain containing proteins (POPDC1/2/3) interact with TREK-1, leading to an augmentation of current that results from enhanced expression of TREK-1 at the cell surface [76]. Recent work has also implicated TREK-1 in the regulation of hypertrophic growth under stress, with a cardiac-specific knockout of TREK-1 resulting in increased activation of the transcription factor STAT3 and concomitant structural remodeling. Stress-activated kinases have also been reported to be affected in this model, with the loss of TREK-1 leading to a reduction in JNK/c-Jun activity [77]. This may contribute to the maladaptive impact of TREK-1 loss in the heart by reducing cardioprotective feedback [78]. Overall, TREK-1 functions as an integrator of stress signaling in cardiac myocytes, tuning the ionic balance – and more recently, the nonionic intracellular signaling – to maintain cellular function during stress.

## Fibroblasts

Fibroblasts are non-excitable cells that play a role in maintaining the extracellular matrix (ECM) and stabilizing the mechanical and immunologic microenvironment of many organ systems [79]. While not a classical excitable cell like myocytes or neurons, fibroblasts express many proteins that regulate and respond to membrane potential and influence cellular behavior [80]. In this context, it is interesting that TREK-1 operates in fibroblasts as an integrative mechanotransducer that links membrane tension, lipid signals, and environmental stresses to Ca^2+^ entry, MAPK activation (JNK, ERK, p38), fibroblast differentiation, fibrosis, ECM deposition, and tissue stiffening.

Mechanosensation is a well-studied phenomenon in fibroblasts given their role in ECM homeostasis and structural remodeling. Piezo channels are one of the most well-studied families of biological mechanosensors and have been shown to functionally couple with TREK-1 and other K_2P_ channels [81]. In the case of TREK-1, this interaction does not significantly alter the single channel current, but instead increases the number of active channels at the membrane [82]. Similar to TREK-1, Piezo channels open when cells experience mechanical stress, allowing influx of cations that alter membrane tension and lipid composition, promoting TREK-1 activation. Thus, Piezo channels act as mechanical amplifiers for TREK-1, and the combined activity of these channels helps to fine-tune the cell’s response to mechanical stress [82]. Instead of functioning as an isolated stretch-activated channel, in this case TREK-1 is a secondary mechanosensor that integrates/refines signals initiated by primary sensors like Piezo. In this manner, TREK-1 can regulate fibroblast activation even if it is not the primary sensor of force.

Unique among K_2P_ channels, TREK-1 is the only TREK family member that has been identified as being both expressed and functional in cardiac fibroblasts (CFs) with proposed role into regulating cardiac fibrosis with implications for cardiac function [83]. A fibroblast-specific deletion of TREK-1 demonstrated reduced interstitial fibrosis under pressure-overload conditions compared to WT controls [84]. Further, this fibroblast-specific TREK-1 knockout exhibited reduced JNK and c-Jun phosphorylation after mechanical stretch, TGF-β or EGF stimulation in isolated CFs, demonstrating the importance of TREK-1 in activating these pathways [84]. The authors do not comment on possible mechanisms for this regulation, but these mirror other findings in alternate cell types in which TREK-1 exerts regulatory effect on pathways not understood to be electrically regulated [77]. This unexpected regulatory capacity prompts exciting questions regarding the mechanisms and implications of TREK-1 activation and dysregulation.

Fibroblast-like synovial cells have been found to express TREK-1 and respond to arachidonic acid stimulation [30]. This leads to cell hyperpolarization and modifies calcium signaling, with downstream effects on cellular growth, cytokine secretion and inflammation. Inhibition of TREK-1 by the selective blocker spadin prevents this activation, identifying TREK-1 as a mediator between fatty acid signaling and calcium signaling in fibroblast-like synovial cells, highlighting its role as a potentially important player in joint diseases like rheumatoid arthritis, while also highlighting the significance of TREK-1 as beyond just a contributor to electrical regulation [30].

In a model of idiopathic pulmonary fibrosis (IPF) TREK-1 activity was found to contribute to progression of disease, with pharmacologic knockdown of the channel attenuating fibrosis [85]. The authors discover a complex intercellular regulation between pulmonary fibroblasts and alveolar macrophages underpinned by TREK-1. The authors did not discuss differential expression of TREK-1 in fibroblasts, but confirmed using human data that TREK-1 is upregulated in pulmonary tissue from IPF patients, suggesting modulation of TREK-1 May be a viable therapeutic strategy.

Hepatic stellate cells (HSCs), another fibroblast-like cell shown to express TREK-1, can transdifferentiate into myofibroblast-like cells and generate liver fibrosis [86]. In human hepatic stellate LX-2 cells, a model of activated HSCs, silencing TREK-1 suppresses cell proliferation and the expression of collagen I. Similar to its activity in CFs, in HSCs TREK-1 inhibition reduces phosphorylation of ERK1/2 and JNK, MAPK signaling pathways that promote fibroblast proliferation and ECM production, making TREK-1 an upstream regulator of MAPK-dependent fibrotic signaling. Thus, TREK-1 activity helps maintain the pro-growth, pro-fibrotic phenotype of these cells, effects underscoring its function beyond that of a typical ion channel [86].

Across organ systems, the activation of fibroblasts supports recovery from injury, stabilizing the mechanical microenvironment while tissues recover. These studies highlight the conserved relationship TREK-1 has with remodeling processes, with its activity in fibroblasts and fibroblast-like cells generally leading to promotion of fibrosis, despite differential activation pathways in these cell types.

## Immune cells

Immune cells form a much more diverse group of cells than those previously discussed. Expression of TREK-1 has not been identified in all immune cell types, but has been confirmed in several macrophage subtypes, and neutrophils [87–89]. In these cell types, TREK-1 has been identified as a crucial mediator of response to injury, often by mechanisms that remain unresolved. TREK-1 is highly associated with several inflammatory responses particularly in hyperoxia or infection mediated lung injury. Increased expression of TREK-1 is observed in lung tissues with bleomycin (BLM)-induced pulmonary fibrosis [85]. Overexpression of TREK-1 in macrophages promotes polarization to M2 (anti-inflammatory) phenotype, profibrotic factor production and enhanced trans differentiation of lung fibroblasts into myofibroblasts (through TGFβ1 expression) [85]. Increased TREK-1 expression inducing M2 polarization is also observed in lung macrophages from IPF patients [85]. Studies describing the role of TREK-1 in modulating temporal changes in macrophage phenotype during disease conditions and healing process would further aid in designing efficacious therapies.

In addition to modulating macrophage gene expression and activity, TREK-1 is also crucial for activation of the inflammasome in macrophages. The NLRP3 inflammasome is a key inflammatory regulator, involved in the pathogenesis of viral and bacterial infections [90,91]. Inflammasome signaling requires both priming and activation steps, in which K^+^ efflux is crucial [92,93]. In alveolar macrophages, TREK-1 deficiency is shown to abrogate LPS-induced secretion of IL-1β and pro-caspase-1 cleavage, decreasing inflammasome activation. TREK-1 deficiency was not shown to affect inflammasome priming. TREK-1-mediated modulation of intracellular K^+^ concentration in alveolar macrophages facilitates activation of the NLRP3 inflammasome by downstream mechanisms that are not fully understood [87]. These studies have shown that the action of TREK-1 contributes to the response to hyperoxia induced lung injuries. This may combine with the expected enhancement of infiltration of inflammatory cells including macrophages and neutrophils in the setting of increased STAT3 levels [77,94].

In the context of disease progression TREK-1 is also associated with secondary degeneration leading to neurological impairment and injury post intracerebral hemorrhage (ICH). In a surgical model of ICH, TREK-1 deficiency was shown to aggravate immune cell infiltration, microglia activation and secretion of proinflammatory chemokines and cytokines, worsening outcomes [89]. In spinal cord injuries TREK-1 deficiency augments focal inflammatory reactions [89]. Apart from regulating immune functions in mouse models of experimental autoimmune encephalomyelitis (EAE), TREK-1 activation by alpha linolenic acid was found to impede activation of microglia, reducing apoptosis and supporting functional recovery in a model of cerebral ischemia [95,96]. These findings contrast with the previously discussed effects of TREK-1 in pulmonary inflammatory cells, raising questions about the downstream effects of TREK-1 activation and the differential activity of its role in the inflammasome. These highlight the broad range of responses that TREK-1 has demonstrated involvement in, and the consistent theme that the presence of TREK-1 supports a balanced immune response.

## Other cells

In addition to the groups previously discussed, TREK-1 has been identified and studied in other cell types. For lack of clear grouping, those findings are discussed here.

In adipocytes, TREK-1 plays an important role in regulating adipogenesis, the process by which preadipocytes differentiate into mature adipocytes. During adipogenesis, TREK-1 expression declines significantly, altering the membrane potential and allowing intracellular calcium levels to increase through voltage-dependent calcium channels (VDCCs). In vitro and ex vivo experiments have consistently demonstrated that inhibiting TREK-1 promotes adipogenesis, while activating TREK-1 inhibits adipogenesis. The study also showed that TREK-1 knockout mice on a high-fat diet displayed more adipogenic properties, including greater fat mass and insulin resistance. Together, these findings suggest that TREK-1 deficiency plays an important role in the development of obesity and that TREK-1 activation can be an important therapeutic target to slow down adipogenesis [97].

TREK-1 was also found to play an important role in endothelial cells of mesenteric (and cutaneous) arteries. In vivo experiments in mice have shown that mice with a global TREK-1 knockout experience less vasodilation than wildtype mice in response to acetylcholine (ACh) and bradykinin (BK). However, no difference in vasodilation of isolated mesenteric arteries was found between the wildtype and TREK-1^−/-^ mice after inhibition of the synthesis of NO, or after exposure to the calcium ionophore A23187, which causes influx of calcium into the endothelium without the use of receptors. Although the precise mechanism is unclear, these results suggest that TREK-1 is likely involved in the signaling pathway (at a step between G-protein activation by vasodilators and calcium increase in the cell) that activates NO synthesis [98].

Compared to adipocytes and endothelial cells, TREK-1 has been studied extensively in various epithelial cells across a range of diseases. Studies on alveolar epithelial cells (AECs) have uncovered a role for TREK-1 in regulating cytokine secretion and stretch-induced cell detachment. TREK-1 treatment of mouse and human epithelial cell lines with TREK-1 shRNA results in higher MCP-13 (CCL2) and lower IL-64 secretion. In a separate experiment, TREK-1 deficient AECs were found to have less of the cytoskeletal protein F-actin, rendering them more deformable and more resistant to injury from stretch [99]. However, subsequent experiments found no link between their cytokine secretion and cytoskeletal rearrangements [100]. More recent in vitro and in vivo experiments involving human cell lines and mice demonstrated the therapeutic potential of the novel TREK-1 activators ML335 and BL1249 in hyperoxia- and influenza A-induced lung injury. In both cases, treatment with ML335 and BL1249 decreased secretion of the pro-inflammatory cytokines IL-6, IP-10, and CCL2 by AECs, increased TREK-1 K^+^ currents in AECs, and improved broncho-alveolar lavage protein levels and cell counts, counteracting the effects of the two diseases [101–103]. Elevated TREK-1 expression was observed in the human prostate cancer cell lines PC3 and LNCaP and the human ovarian cancer cell lines SKOV-3 and OVCAR-3 compared to healthy prostate and ovarian epithelial cells [104,105]. Adenoviral transduction experiments probing this finding using adTREK-1 found that TREK-1 overexpression increased cell proliferation in normal prostate epithelial (NPE) cells and Chinese hamster ovary cells [105]. The reasons underlying the observed increase in TREK-1 expression in cancer cells remain unanswered, but changes in lipid metabolism from the altered expression of 15-lipoxygenase (an enzyme that converts polyunsaturated fatty acids to various biologically active molecules) and changes in intracellular pH are suggested as possible factors [104–106]. Experiments have shown that TREK-1 blockers can inhibit cell proliferation and affect apoptosis in NPE cells and the ovarian cancer cell lines, suggesting that TREK-1 could be a possible therapeutic target in prostate and ovarian cancer [104,105].

TREK-1 has also been identified in the gastrointestinal tract, in the epithelial lining, and smooth muscle cells (SMC) particularly of the stomach and colon where it is believed to contribute to mechanical feedback [107–109]. Particularly interesting is the finding of differential expression in Hirschsprung disease (HSCD). In both aganglionic and ganglionic bowel sections, TREK-1 expression is reduced compared to healthy control tissue [108]. TREK-1 has also been identified as crucial in regulation of intestinal barrier integrity, further supporting its relevance in HSCD pathology [109].

## Conclusion

TREK-1 is widely expressed in the central nervous system, heart, and peripheral tissues [9]. In the cardiovascular system, TREK-1 expression has been confirmed in atrial and ventricular cardiomyocytes, where it exhibits a transmural gradient with higher expression in the endocardium compared to the epicardium [32]. Physiologically, TREK-1 contributes to the maintenance of the resting membrane potential and the repolarization of the action potential [38,65]. Due to its mechanosensitivity, TREK-1 is hypothesized to play a key role in cardiac mechano-electric feedback, potentially counterbalancing inward currents from stretch-activated nonselective cation channels to prevent arrhythmias during mechanical stress [32,110]. Additionally, TREK-1 has been implicated in the regulation of sinoatrial node excitability; cardiac-specific deletion of TREK-1 leads to bradycardia and sinus pauses following stress [74].

The channel’s role extends to pathology, where its expression and function are altered. TREK-1 is upregulated in cardiac hypertrophy and downregulated in atrial fibrillation and heart failure [32,110]. Furthermore, specific mutations in TREK-1 that alter its ion selectivity, allowing sodium influx, have been linked to right ventricular outflow tract tachycardia [17,39]. TREK-1 has been implicated as a regulatory component in the disease processes of many tissues, both excitable and non-excitable, showcasing a remarkably diverse range of activation and effector modalities. The channel integrates discrete stimuli from its electrical, mechanical, and chemical microenvironment to potentiate an equally broad range of effects to regulate not only membrane potential, but also intracellular signaling, gene expression, cytoskeletal remodeling, and cell–cell communication. Although it is certain that in part some of the regulatory effects of TREK-1 are related to its action as a transmembrane ion channel, this confluence of nonionic regulatory activity suggests that it may play a greater role. While individual sections of this review highlight cell-specific consequences of TREK-1 modulation, the shared mechanistic motifs – MAPK activation, Ca^2+^ handling, mechanotransduction, and remodeling – emerge consistently. These cross-tissue roles illustrate how a single channel mediates strikingly diverse physiological and pathological processes. This cross-cell comparison underscores TREK-1’s unique position as a convergent signaling hub and highlights why perturbations in its regulation have such broad systemic implications. Perhaps TREK-1 expression is subject to even more regulation, enhancing or suppressing its activity in pathological contexts invisible to the models presented here. Alternately, its intracellular domains may contain crucial regions for protein-protein interaction, serving to link other regulatory effectors with their targets. Consequently, TREK-1 has emerged as a potential therapeutic target for cardiovascular diseases, as well as for pain, depression, and ischemia, prompting the development of novel pharmacological modulators such as the inhibitors L-3-n-butylphthalide (L-NBP) and spadin, and various activators such as ML335 [30,32].

## Data Availability

This review article does not include any original data. All information discussed is derived from previously published studies and publicly accessible sources available online. No new datasets were generated or analyzed for this work. All referenced materials can be accessed through their respective publishers or repositories as cited in the manuscript.
